# Anti-central fatigue effects of myelophil in 5-HTergic hyperactivity mice model

**DOI:** 10.1186/s12906-025-04882-2

**Published:** 2025-04-23

**Authors:** Ji-Yun Kang, Dong-cheol Baek, Jin-Seok Lee, Chang-Gue Son

**Affiliations:** 1 Institute of Bioscience & Integrative Medicine, Collage of Korean Medicine, Daejeon University, 22–5 Daedukdae-ro 176 beon-gil 75, Seo-gu, Daejeon, Republic of Korea; 2 Research Center for CFS/ME, Daejeon University Hospital, 22–5 Daedukdae-ro 176 beon-gil 75, Seo-gu, Daejeon, Republic of Korea

**Keywords:** Central fatigue, Serotonin, Myelophil, ME/CFS, Fatigue

## Abstract

**Background:**

Myelophil is a standardized ethanol extract of *Astragali Radix* and *Salviae Miltiorrhizae Radix*, which has been developed based on clinical experience in traditional Korean medicine practices for patients with unexplained chronic fatigue, including myalgic encephalomyelitis/chronic fatigue syndrome (ME/CFS). Our previous studies demonstrated Myelophil’s clinical efficacy in ME/CFS, as well as its brain-related activities in animal models. However, the underlying pharmacological mechanisms remain unclear. Recently, we identified serotonergic hyperactivity as a key pathophysiological factor in central fatigue, such as ME/CFS. Therefore, in the present study, we aimed to investigate the mechanisms by which Myelophil exerts its effects, particularly in the context of a 5-HTergic hyperactivity model.

**Method:**

To verify the action mechanisms of Myelophil on serotonergic hyperactivity condition, we herein assessed its anti-central fatigue properties using a fluoxetine-treated mice model. Male C57BL/6 N mice (9 weeks old) were subjected to periodic intraperitoneal (IP) injections of fluoxetine for 4 weeks and the mice were simultaneously oral administered Myelophil (0, 50, or 100 mg/kg) or ascorbic acid (100 mg/kg).

**Result:**

Four-week injection of fluoxetine notably increased serotonin (5-hydroxytryptamine, 5-HT) activity, as evidenced by immunofluorescence staining and Western blot assays in the raphe nuclei (RN), and induced central fatigue-like behaviors in the nest building test, wheel running test, rota-rod test, plantar test, and open field test. Meanwhile, Myelophil (100 mg/kg) administration significantly ameliorated those fatigue-related behaviors including pain sensitivity. Furthermore, the anti-fatigue effects of Myelophil were corroborated by changes in serotonin-related parameters (serotonin transporter; 5-HTT and vesicular monoamine transporter 2; VMAT2), as well as neurotrophic markers including c-Fos and brain-derived neurotrophic factor (BDNF) in the RN.

**Conclusion:**

These results provide experimental evidence suggesting the potential mechanisms by which Myelophil may alleviate central fatigue associated with hyper-5-HTergic activity.

**Clinical trial number:**

Not applicable.

**Supplementary Information:**

The online version contains supplementary material available at 10.1186/s12906-025-04882-2.

## Introduction

Fatigue, functioning both as a physiological defense mechanism and a symptom associated with various diseases, is a prevalent issue among patients with diverse disorders as well as the general population [1]. Fatigue can be classified by its duration into acute (≤ 1 month), prolonged (1 to ≤ 6 months), and chronic (lasting over 6 months) [2], and it can also be divided into medically explained and unexplained fatigue [3]. Chronic fatigue has significant clinical implications, affecting an estimated 7.7% of the general population and approximately 50% of cancer patients [4]. In particular, medically unexplained chronic fatigue more adversely affects the health-related quality of life (QOL), including myalgic encephalomyelitis/chronic fatigue syndrome (ME/CFS) [5].

Fatigue can also be categorized as either peripheral or central. Central fatigue refers to pathogenic conditions related to neuromuscular dysfunction, primarily resulting from biochemical alterations in the brain [6]. In contrast to peripheral fatigue, which primarily affects muscles due to energy-related disruptions, central fatigue results from synaptic transmission dysfunction in the central nervous system (CNS) [7]. Along with multiple sclerosis and post-stroke syndrome, especially ME/CFS is considered a typical disorder associated with central fatigue, characterized by muscle weakness and poor concentration [8, 9]. The most prominent hypothesis regarding the pathophysiological mechanisms of central fatigue is the imbalance between serotonin (5-hydroxytryptamine, 5-HT) and dopamine (DA) in the brain [10].

Generally, 5-HT plays a key role in various physiological and psychological functions of the brain [11]. Low levels of 5-HT are widely recognized as the cause of a variety of brain disorders, including depression, anxiety, psychosis, and other health conditions [12]. Conversely, elevated 5-HT levels can result in central fatigue [13]. Some clinical observations have led to the proposal of the ‘serotonin hypothesis’ as a potential pathophysiological mechanism of central fatigue [14]. Additionally, other clinical studies have shown that selective serotonin reuptake inhibitors (SSRIs), a class of antidepressants, reduced the overall movement in subjects due to central fatigue by affecting neurotransmitters in the CNS [15, 16]. We recently demonstrated that limbic hyper-5-HTergic activity could lead to ME/CFS-like behaviors using an animal model with both SSRI injection and viral vector-derived inhibition of 5-HT_1A_ receptor (5-HT_1A_R) [17]. Consequently, numerous research groups have begun investigating conditions associated with neurotransmitter alterations as potential targets for central fatigue disorders [18, 19].

Myelophil, a 30% ethanolic extract consisting of equal parts of *Radix Astragali* and *Radix Salviae Miltiorrhizae*, is a commercially available supplement in Korea, commonly used for treating fatigue-associated disorders [20]. Previous investigations into the pharmacological properties of Myelophil have demonstrated its potent anti-muscle fatigue, anti-neuroinflammatory, and antioxidative effects, among others [21–25], and confirmed its genotoxicity using a bacterial reverse mutation test [26], as well as acute and repeated toxicological studies in beagle dogs [27]. In addition, our phase 2 clinical study also showed therapeutic effect for patients with ME/CFS [28]. However, the mechanical studies for Myelophil on central fatigue, especially under the model of hyper-5-HTergic activity, have not yet been investigated.

The purpose of this study was to examine the anti-central fatigue effects of Myelophil and elucidate its underlying mechanisms using a fluoxetine-induced hyper-5-HTergic activity mouse model.

## Methods

### Preparation and standardization of myelophil

**Fig. 1:**
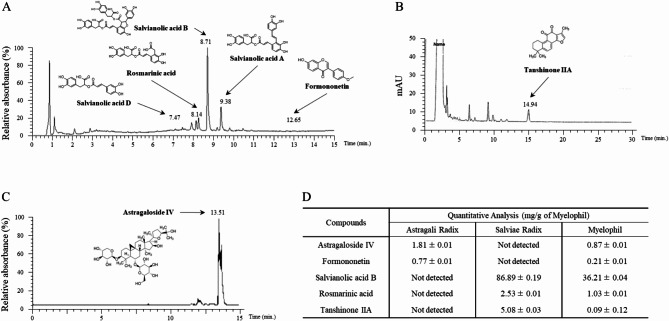
Fingerprinting analysis of Myelophil (MYP). Formononetin, salvianolic acid B, rosmarinic acid (**A**), tanshinone IIA (**B**) and astragaloside IV (**C**) using UHPLC. Myelophil and five major compounds were analyzed by LC/MS (**D**)

*Astragali Radix* (Astragalus membranaceus Bunge, cultivated in Jecheon, South Korea; Ser. No. 20101106-JC-HG) and *Salviae Miltiorrhizae Radix* (Salvia miltiorrhiza Bunge, cultivated in Hebei, China; Ser. No. 20110302-CHN-DS) were purchased from an Eastern medicine company (Jeong-Seong Drugstore, Daejeon, Korea). Equal proportions of *Astragali Radix* and *Salviae Miltiorrhizae Radix* were extracted using 30% ethanol, and formulated into Myelophil (Kyung-Bang Pharmacy, Incheon, South Korea). It was produced in accordance with the approved good manufacturing practice (GMP) guidelines of the Korean Food and Drug Administration (KFDA). The final Myelophil product [yield: 20.52% (w/w)] was stored for future use. Molecular fingerprinting of Myelophil (Fig. 1A to C) was conducted by ultra-high-performance liquid chromatography (UHPLC, Thermo Scientific, San Jose, CA, USA) as described previously [29]. The quantitative analysis (Fig. 1D) of four reference compounds (Aastragaloside IV and formononetin for *Astragali Radix*, and salvianolic acid B, Rosmarinic acid, and Tanshinone IIA for *Salviae Miltiorrhizae Radix*) and Myelophil was performed using liquid chromatography-mass spectrometry (LC/MS, LTQ Orbitrap XL linear ion-trap MS system, Thermo Scientific Co., San Jose, CA, USA), as described previously [24].

### Animal and experimental design

Forty male C57BL/6J mice, 9 weeks old, weighting 22–25 g, were procured from Dae Han Bio Link (Co., Ltd., Eumseong, Korea). These mice were devoid of specific pathogens. The mice were confined in a room that was maintained at 23 ± 1 °C and followed a 12 h:12 h light–dark cycle. They provided with *ad libitum* access to water and diet pellets (Cargill Agri Purina, Seongnam, Korea). The animal care and experimental protocols were approved by the Institutional Animal Care and Use Committee of Daejeon University (DJUARB2023-038) and conducted in accordance with the guide for the care and use of laboratory animals published by the U.S. National Institutes of Health (NIH). Mice were employed in experiments after a seven-day period of acclimation.

Following a 7-day acclimation period, we randomly divided the mice into five groups, each containing eight mice: saline (saline intraperitoneal; IP injection and distilled water oral administration), Flx (fluoxetine IP injection; 20 mg/kg and distilled water oral administration), MYP 100 and 50 (fluoxetine IP injection and Myelophil oral administration; 100 or 50 mg/kg), and positive control (fluoxetine IP injection and ascorbic acid oral administration; AA, 100 mg/kg). Fluoxetine and Myelophil were dissolved in saline and distilled water, respectively. We injected fluoxetine, a representative SSRI (5-HT enhancer), via injection for 4 weeks, using a dose for mice that was substantially higher than the clinical dose (2 to 5 times greater) [17].

### Nest building test

General malaise and sickness behavior of rodents were evaluated using pressed cotton squares (Envigo, IN, USA) [30] and it can also be useful for testing movement disorders [31]. Briefly, a total of 12 g of pressed cotton squares (ten squares per cage, 7 × 5 cm) were positioned in the center of the floor of a cage that housed eight mice. The mice were scored on a scale of 0 to 5 based on the extent to which they bit the squares, moved them into the corners, and nested with the squares overnight.

### Wheel running test

Exercise capacity was evaluated using motor-driven running wheel equipment (YLS-B10, Yiyuan Technology Development Co., Shandong, China) according to the manufacturer’s instructions with slight modifications [17]. Briefly, the exercise procedure consisted of an electric charge of 2.0 mA and a progressive increase in velocity from 15 to 30 rpm. If a mouse ceased running for more than 2.5 s, despite receiving shocks, the rotating wheel was halted for 30 s. This procedure was repeated three times, at which point the mice were deemed to be experiencing physical exhaustion. Two parameters—exercise duration and distance traveled—were employed to assess exercise tolerance.

### Rota-rod test

Motor coordination and balance was evaluated using a rotarod machine (ENV-574 M, Med Associates Inc., VT, USA) following established research protocols with slight modifications [32]. Briefly, the mice were habituated to an accelerating speed from 2 to 20 rpm for 560 s. During habituation, the mice were immediately placed back on the drum at most five times after they fell to the bottom of the apparatus. The motor activity and sense of balance were evaluated by the latency to fall during drum acceleration from 4 to 40 rpm for 300 Sect. (5 trials interval 20 min), respectively.

### Plantar test

Thermal pain sensation in rodents was determined using a Hargreaves apparatus (37370-002, Ugo Basile, Comerio, Italy) following established research protocols with slight modifications [33]. Briefly, for a period of 15 min, each mouse was confined to a plexiglass cubicle with measurements of 8.5 cm in length, 3.4 cm in width, and 3.4 cm in height. The plantar surface of the hind paw was exposed to a radiant heat source with a constant intensity of 70 I.R. The latency to paw withdrawal was documented.

### Open field test

Anxiety and locomotion were evaluated using a large square chamber (length 40 × width 40 × height 30 cm) following established research protocols with slight modifications [34]. Briefly, the center area (25 × 25 cm) was designated in the recording software. The rodents were permitted to freely investigate the field for 5 min after acclimation for 30 min in a testing chamber with 50 lx illumination under red light. The duration of time spent in the central area was documented.

### Blood collection and brain tissue Preparation

Mice were sacrificed under CO_2_ anesthesia on the last day of the fluoxetine injection. Blood samples from five mice per group were collected according to the instructions of the Animal Management and Use Committee. After centrifugation at 3000 rpm for 15 min at 4 °C, we collected the serum and stored it at -80 °C. We immediately isolated the raphe nuclei (RN) from the whole brain of the five blood-collected mice and stored the samples at -80 °C. We then pooled five mouse brains in RIPA buffer to homogenize for biochemical analyses, such as enzyme-linked immunosorbent assay (ELISA) and western blotting, and assessed the pooled brain samples in triplicate. Following transcranial perfusion, we fixed the brains of three mice per group, not used for blood collection in a 4% paraformaldehyde (PFA) solution for immunofluorescent staining.

### Measurement of protein concentration for biochemical analysis

Protein concentrations of brain sample was determined the using a Bicinchoninic Acid (BCA) protein assay kit (Catalog No. 23227, Thermo Fisher). Briefly, 25 µL of samples or standards were pipetted into a 96-well microplate. Reagents A and B were mixed at a 50:1 ratio to create the working reagent, and 200 µL of this reagent was added to each sample or standard. The plate was incubated at 37 °C for 30 min, and absorbance at 562 nm was measured using a UV spectrophotometer (Molecular Devices).

### Determination of the corticosterone level in serum

The manufacturer’s instructions (Catalog No. K014-H5, Arbor Assays, Michigan, USA) were followed to measure the serum level of corticosterone using commercial ELISA kits. A UV spectrophotometer (Molecular Devices) was employed to measure the absorbance at 450 nm.

### Immunofluorescence staining analysis for 5-HT

The day after the last day of fluoxetine injection, the mice were transcranial perfused with 0.05% heparin (10 units/mL in PBS), followed by 4% PFA (pH 6.9). The brains that were removed were cryoprotected in 10%, 20%, and 30% sucrose for 24 h each. Subsequently, they were embedded in an optimal cutting temperature (OCT) compound (Leica Microsystems, Bensheim, Germany) in liquid nitrogen. They were cut into frozen coronal Sect. (30 μm) using a cryostat (CM3050_S, Leica), and sections were stored in free-floating buffer. After incubating with blocking buffer (5% normal chicken serum in PBS and 0.3% Triton X-100 for 1 h at 4 °C), section slices, including dorsal raphe nuclei (DRN), were adapted with anti-goat 5-HT (1: 100, ab66047, Abcam, Cambridge, MA, USA) primary antibodies overnight at 4 °C. After washing with ice-cold PBS, the sections were incubated with anti-goat (1:400; Alexa Fluor 488, ab150129) secondary antibodies for 2 h at room temperature. The sections were subsequently exposed to 4,6-diamidino-2-phenylindole dihydrochloride (DAPI) to stain the cell nuclei. Immunofluorescence images were captured under an Axio photo microscope (Carl Zeiss, Jena, Germany), and the signals were quantified using ImageJ 1.46 software (NIH, Bethesda, MD, United States).

### Western blotting analysis

The lysates of brain tissue were separated by polyacrylamide gel electrophoresis and transferred to polyvinylidene fluoride (PVDF) membranes. After the membranes were blocked in 5% skim milk in tris buffered saline (TBST; 0.05% Tween 20 in TBS) for 1 h, they were probed with primary antibodies against 5-HT transporter (5-HTT; 1:1000, AB9726, Merck, Darmstadt, Germany), tryptophan hydroxylase 2 (TPH2; 1:1000, NB300-74555, Novus, St. Louis, MO, USA), vesicular monoamine transporter 2 (VMAT2; 1:1000, NB110-68123, Novus), monoamine oxidase type A (MAO-A; 1:1000, ab126751, Abcam), 5-HT_1A_R (1:1000, ab85615, Abcam), tropomyosin receptor kinase B (TrkB; 1:1000, sc-377218, Santa Cruz, Dallas, TX, USA), cAMP response element-binding protein (CREB; 1:1000; ab31387, Abcam), phospho-CREB (*p*-CREB; 1:1000; ab108319, Abcam) brain-derived neurotrophic factor (BDNF; 1:1000; ab108319, Abcam), c-Fos (1:1000, ab208942, Abcam) and α-tubulin (1:1000, ab7291, Abcam) overnight at 4 °C. The membranes were washed and incubated with HRP-conjugated anti-mice (1:5000, against TrkB, c-Fos, and α-tubulin) or anti-rabbit (1:5000, against 5-HTT, TPH2, VMAT2, MAO-A, 5-HT_1A_R, CREB, *p*-CREB, and BDNF) antibodies for 45 min. The protein was visualized using an enhanced chemiluminescence (ECL) kit. Protein expression was observed using the FUSION Solo System (Vilber Lourmat, Collegien, France). The intensity was semi-quantified using ImageJ 1.46 software (NIH, Bethesda, MD, USA).

### Statistical analysis

The data are presented as means ± standard deviations. The GraphPad Prism 9 software (GraphPad, Inc., La Jolla, CA, USA) was employed to conduct the statistical analysis. Dunnett’s test was implemented subsequent to one-way variance analysis (ANOVA) to ascertain statistical significance. A significance level of *p* < 0.05 was applied to all analyses.

## Results

### Effects of myelophil on fatigue-like behaviors

**Fig. 2:**
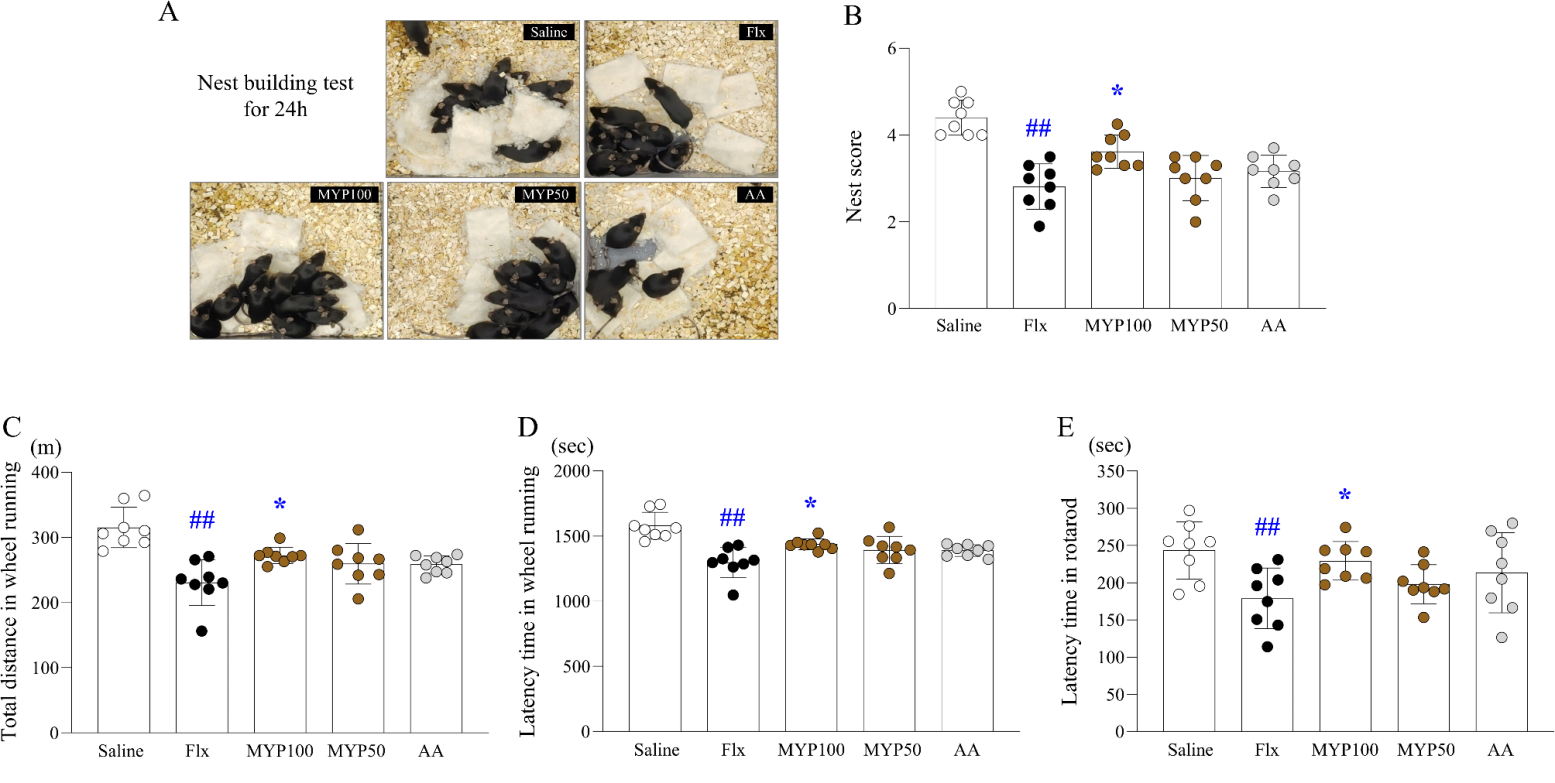
Effects of Myelophil (MYP) on physical fatigue-related behaviors. After fluoxetine (Flx) injection for 4 weeks, the nest-building test was used to assess welfare and general fatigue (**A**), with semi-quantification of the results (**B**). Exercise capacity was investigated by measuring total distance (**C**) and latency time (**D**) in the wheel running test, as well as latency time in the rota-rod test (E). The data are expressed as the means and standard deviations (*n* = 8). ##, *p* < 0.01 compared with the normal group; *, *p* < 0.05 compared with the Flx group

After 4 weeks of fluoxetine exposure, fatigue-like behaviors were assessed using three experiments, including the nest building test, the rota-rod test and the wheel running test. As expected, mice showed fatigue-like behaviors by fluoxetine treatment compared with the saline group (*p* < 0.01). However, these behaviors were attenuated by the administration of Myelophil (in particular 100 mg/kg), as evidenced in the nest score in the nest building test (*p* < 0.05, Fig. 2A and B), total distance in the wheel running test (*p* < 0.05, Fig. 2C), and latency time in both the wheel running and rota-rod test (*p* < 0.05, Fig. 2D and E). No significant effects were observed with the administration of 50 mg/kg of Myelophil or 100 mg/kg of the AA.

### Effects of myelophil on anxiety and pain sensitivity

**Fig. 3:**
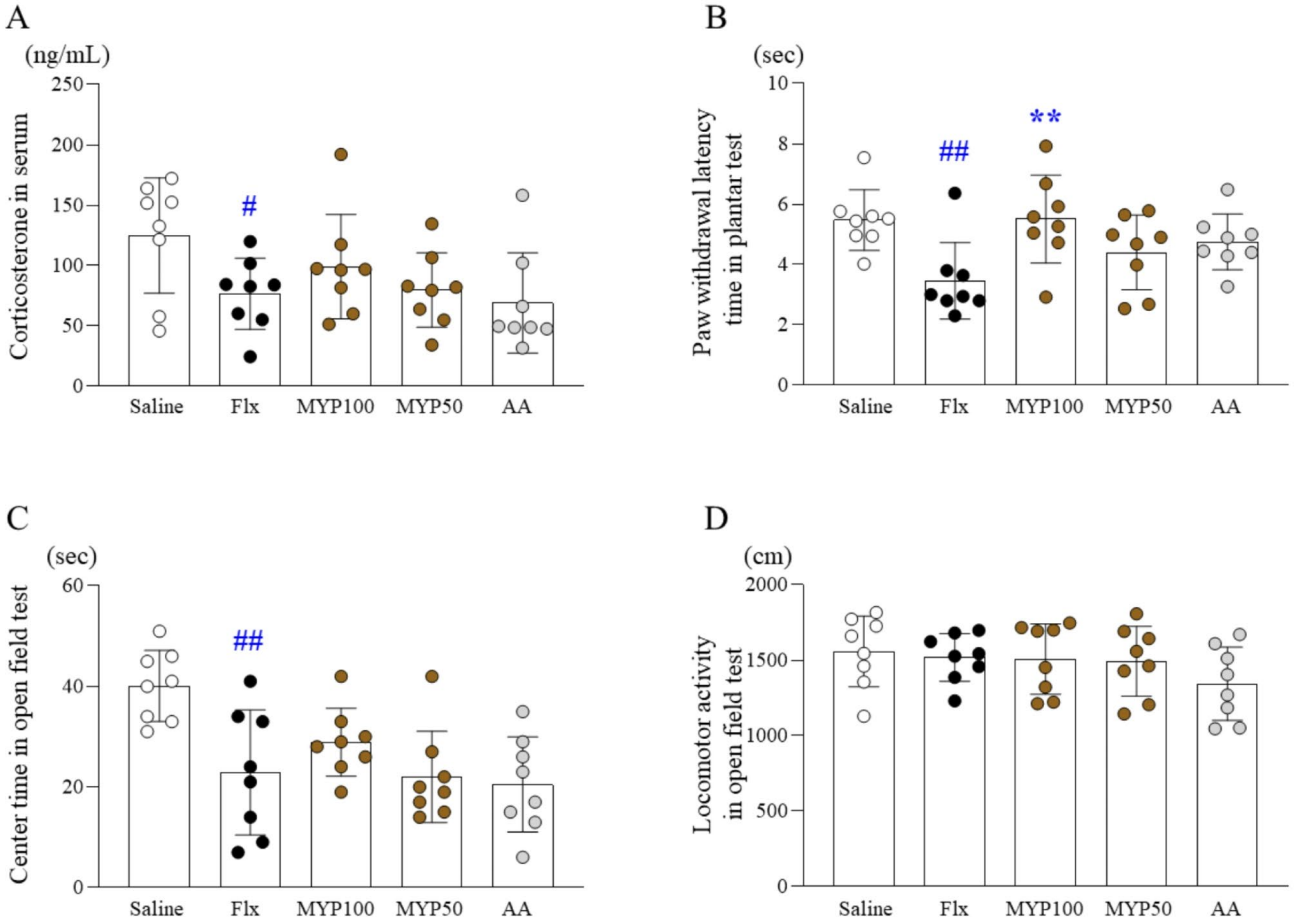
Effects of Myelophil (MYP) on corticosterone, pain-sensitivity, and anxiety behaviors. After fluoxetine (Flx) injection for 4 weeks, serum corticosterone levels were determined using ELISA (**A**), and the plantar test was used to assess latency to paw withdrawal (**B**). Time spent in the center and locomotor activity were assessed in the open field test. The data are expressed as the means and standard deviations (*n* = 3 or 8). #, *p* < 0.05 and ##, *p* < 0.01 compared with the normal group; *, *p* < 0.05 compared with the Flx group

The 4-week fluoxetine injection also induced high pain-sensitivity and anxiety behaviors, as evidenced by a significant reduction in the open field test (latency time in the center zone), and the plantar test (paw withdraw latency time) (*p* < 0.01). Myelophil administration (100 mg/kg) significantly attenuated the high pain-sensitivity (*p* < 0.01, Fig. 3B), while no significant change was observed in anxiety behavior (Fig. 3C and D). No notable changes were observed following the administration of the AA.

### Effects of myelophil on serum corticosterone

The 4-week fluoxetine injection reduced the concentration of serum corticosterone to approximately 0.6-fold compared to the saline group (*p* < 0.05). Myelophil administration showed a tendency toward normalizing this alteration, it did not reach statistical significance (Fig. 3A). No notable changes were observed by the AA administration.

### Effects of myelophil on over-5-HTergic activity in Raphe nuclei

**Fig. 4:**
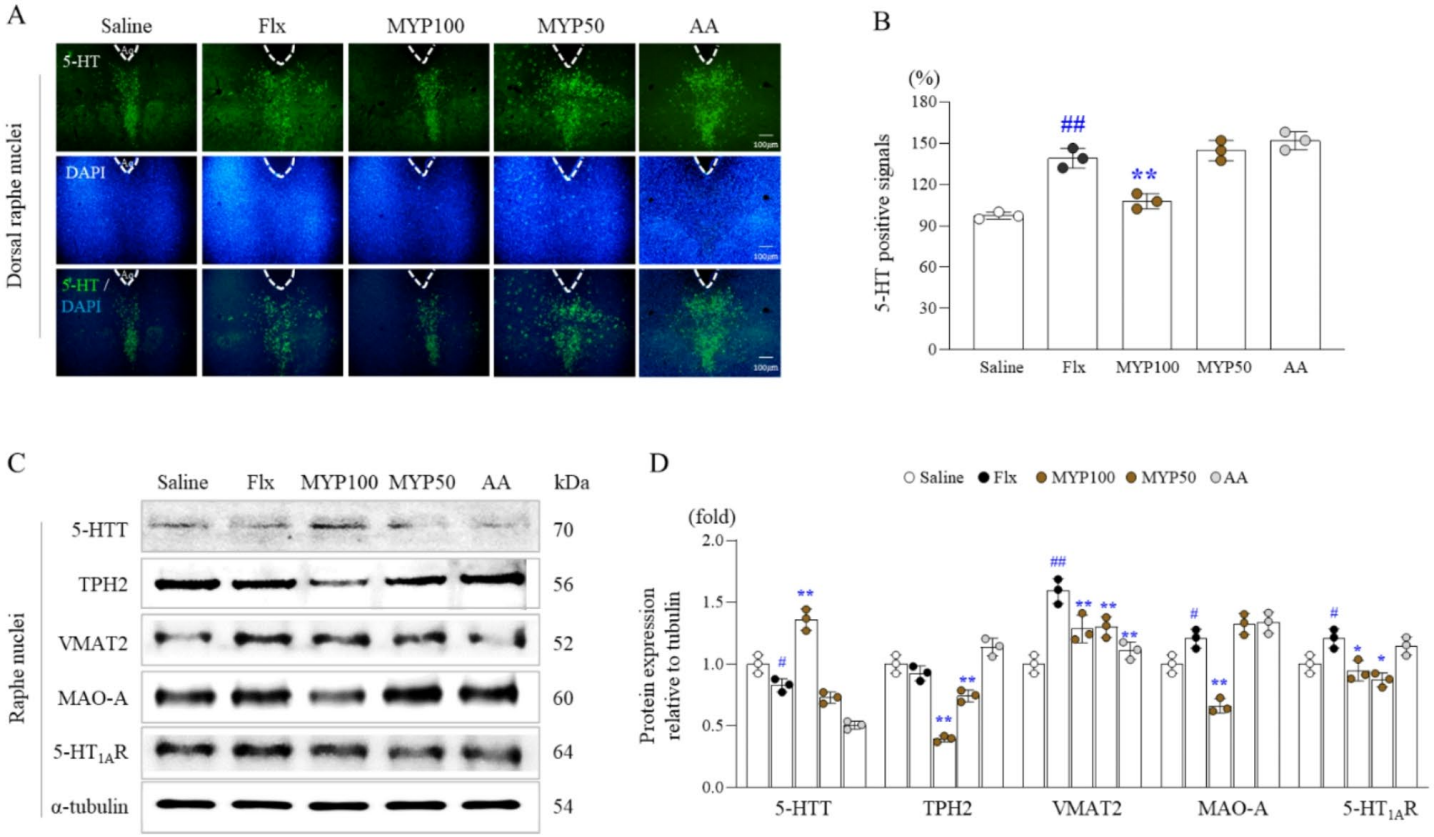
Effects of Myelophil (MYP) on 5-HTergic activity-related molecules in the raphe nuclei (RN). Relative 5-HT levels in the DRN were measured using immunofluorescence (**A**) and semi-quantified (**B**). Western blot analysis was used to assess levels of 5-HTT, TPH2, VMAT2, MAO-A, and 5-HT_1A_R protein expressions in the RN (**C**), with semi-quantification (**D**). The data are expressed as the means and standard deviations (*n* = 3). #, *p* < 0.05 and ##, *p* < 0.01 compared with the normal group; *, *p* < 0.05 and **, *p* < 0.01 compared with the Flx group

As evidenced by immunofluorescence staining for 5-HT, fluoxetine induced overactivity of 5-HT in the DRN by approximately 1.4-fold compared to the saline group, while this alteration was significantly attenuated by the administration with Myelophil (100 mg/kg, Fig. 4A and B). This effect was supported by the measurements of 5-HT-associated molecules as follows. Myelophil administration significantly attenuated the overexpression of 5-HT synthesis/release-regulation molecules (5-HT_1A_R, VMAT2, MAO-A) and increased the depletion of 5-HTT, a molecule for reuptake of 5-HT, respectively (Fig. 4C and D).

### Effects of myelophil on BDNF activity in Raphe nuclei

**Fig. 5:**
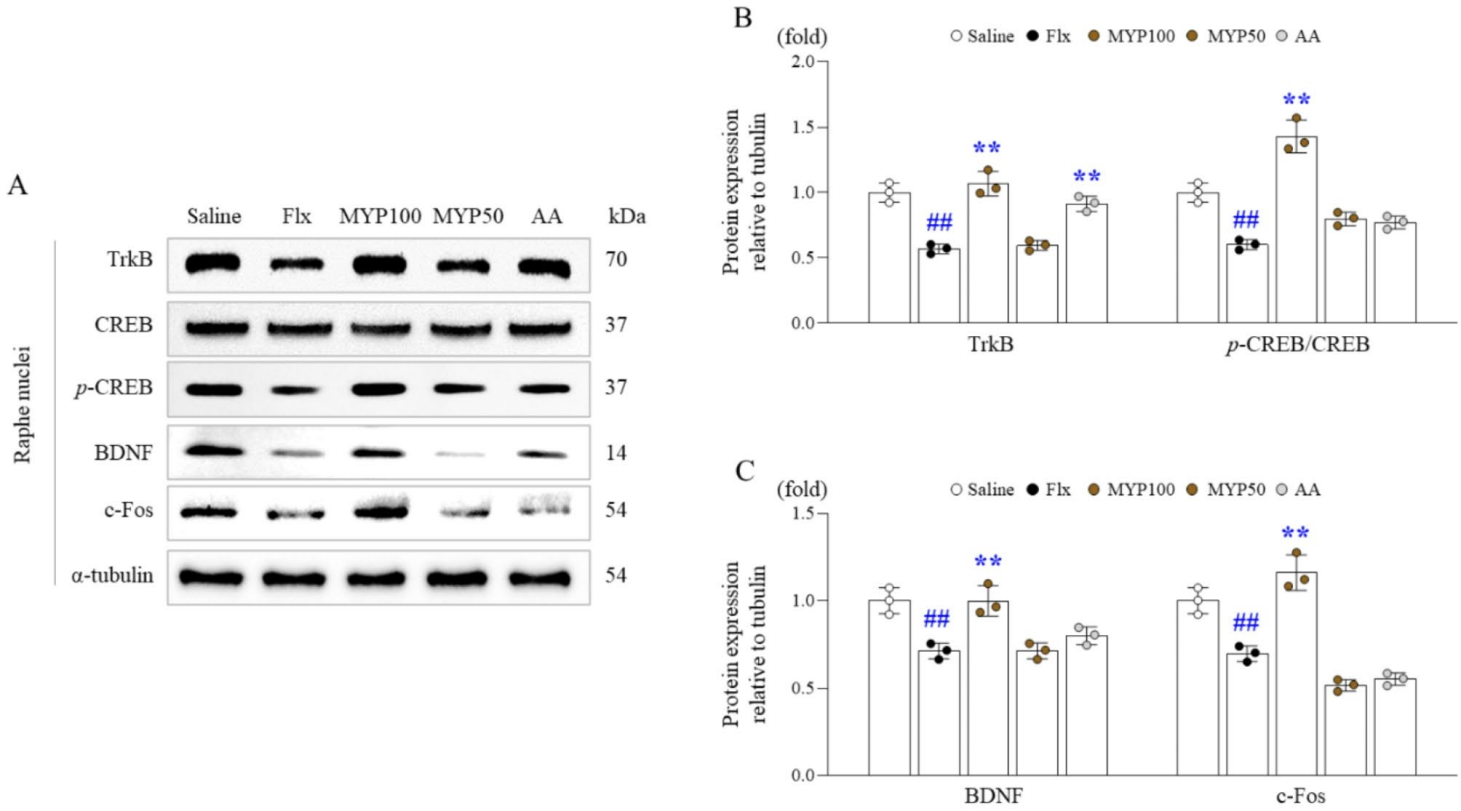
Effects of Myelophil (MYP) on neuronal activity-related molecules in the raphe nuclei (RN). Western blot analysis was conducted to assess levels of TrkB, CREB, p-CREB, BDNF, and c-Fos proteins in the RN (**A**), with semi-quantification (**B** and **C**). The data are expressed as the means and standard deviations (*n* = 3). #, *p* < 0.05, and ##, *p* < 0.01 compared with the normal group; *, *p* < 0.05 and **, *p* < 0.01 compared with the Flx group

The 4-week fluoxetine injection notably suppressed BDNF protein expressions, a typical neurotrophic factor, to approximately half the level observed in the RN. This suppression was significantly reversed by the administration of Myelophil (100 mg/kg). The effect of Myelophil (100 mg/kg) on BDNF was supported by other related molecules including its receptor (TrkB), transcription factor (*p-*CREB), and parameter for neuronal activity (c-Fos), respectively (*p* < 0.01, Fig. 5A to C).

## Discussion

Myelophil, an ethanol extract mixture of *Astragali Radix* and *Salviae Miltiorrhizae Radix*, has been prescribed for patients suffering from fatigue, including ME/CFS patients [28]. It originated from the pharmacological theory focusing on Qi (circulating energy) and blood, two key elementary components in the body according to traditional Korean medicine (TKM) and traditional Chinese medicine (TCM) [35]. Based on our recent finding indicating the crucial pathophysiology of limbic 5-HT hyperactivity in ME/CFS, we herein aimed to assess Myelophil’s effect on 5-HT overactivation-induced central fatigue.

We subjected mice to fluoxetine injection for 4 weeks to induce 5-HT hyperactivity in the brain, which is one of the most widely accepted mechanisms of central fatigue [14]. As we expected, we confirmed that fluoxetine treatment increased 5-HT levels in the RN by approximately 1.4-fold (Fig. 4A and B). Even though there is a conflicting argument, generally, a lower cortisol concentration or reduced cortisol response to stressors is considered a feature of ME/CFS patients [36, 37], as shown in our experiment (Fig. 3A). In contrast to its harmful effects on various systems, including brain and psychiatric disorders [38, 39], cortisol release is a key process to combat stress and is essential to overcoming fatigue-related conditions [40]. As we expected, fluoxetine treatment notably decreased the exercise performance capacity by approximately 0.6-fold (Fig. 2C to E). These changes—5-HT hyperactivity, inhibition of neuronal activity, and lethargy—are all indicative signs of severe central fatigue, which is most likely ME/CFS [41].

ME/CFS is a typical mode of central fatigue, which is linked to alterations in neurotransmitters [42]. Patients with ME/CFS commonly report severe fatigue following daily activities, referred to as post-exertional malaise (PEM), a core symptom in the diagnostic criteria for ME/CFS, along with general malaise without displaying muscular defects [43]. Patients with central fatigue, including ME/CFS, experience reduced force production from their motor units due to muscle membrane dysfunction caused by CNS dysregulation [44]. Myelophil treatment showed improvements in the physical fatigue-associated parameters (rotarod test and wheel-running test), which were exacerbated by fluoxetine (Fig. 2C to E). The execution of rotarod and wheel running assesses the integration of various motor functions, such as balance and muscle endurance [32]. Myelophil has previously demonstrated efficacy in improving physical fatigue scores in ME/CFS patients, assessed using the numerical rating scale (NRS) and the fatigue severity scale (FSS) in randomized clinical trials (RCTs) [28].

Central fatigue manifests with various symptoms that affect not only physical but also mental and emotional health [45]. Patients with ME/CFS frequently show a high comorbidity of pain and anxiety behaviors, with rates ranging from approximately 30 to 70% [46, 47]. In our experiments, fluoxetine exacerbated general motor behavior (measured by the nest-building test), pain sensitivity (plantar test), and anxiety (open field test), while Myelophil administration at 100 mg/kg significantly restored general malaise and sickness behavior (Fig. 2A and B), pain sensitivity (Fig. 3B), and anxiety (Fig. 3C). The nest building test is considered an effective measure of well-being and movement disorders for mice [48], and plantar test evaluates level of pain sensitivity [33]. These results support our previous clinical outcomes, which demonstrated a significant reduction in mental fatigue scores in ME/CFS patients treated with Myelophil for three months compared to placebo [28]. In fact, depression is also one of the most common comorbidities in ME/CFS patients, affecting approximately 30–40% [49]. However, major depressive disorder (MDD) is characterized by decreased 5-HT activity [50], in contrast to our central fatigue model, which presents 5-HT hyperactivity [51]. Several clinical studies have shown that antidepressants, including SSRIs, are ineffective for patients with ME/CFS and sometimes exacerbate fatigue symptoms [52, 53].

Alterations in neurotransmitters, particularly 5-HTergic hyperactivity in the brain, are likely the basis for symptoms in individuals experiencing central fatigue [54]. Human studies have confirmed 5-HT overactivity, with approximately 25% higher levels in ME/CFS patients under the dl-fenfluramine (an indirect central 5-HT agonist) challenge [55, 56]. In our present study, Myelophil administration significantly attenuated fluoxetine-induced overexpression of 5-HT in the DRN (Fig. 4A and B). This finding is supported by clinical observations in ME/CFS patients, which showed reduced 5-HT reuptake in the brain using positron emission tomography (PET) [57]. One study demonstrated that overproduced 5-HT and the activation of 5-HT_1A_R (a representative inhibitory receptor) on the initial axon segments of motor neurons resulted in inhibited neuronal output [7]. Myelophil treatment significantly decreased 5-HT synthesis and release (TPH2, 5-HT_1A_R, VMAT2, and MAO-A) while increasing its reuptake (5-HTT) (Fig. 4C and D). 5-HIAA, the primary metabolite of 5-HT, increases in various brain regions during prolonged exercise and contributes to the onset of fatigue symptoms [58]. These results suggest that Myelophil administration may regulate overall 5-HTergic activity, including serotonin synthesis, transmission, and metabolism, as one of its main mechanisms for controlling central fatigue.

Neurotrophins play a crucial role in regulating the activity of 5-HTergic neurons, and their depletion leads to reduced neuron-to-neuron interactions [59]. Generally, diminished BDNF-TrkB signaling corresponds to reduced 5-HT synthesis and release [60]. Conversely, under conditions of 5-HT hyperactivity, BDNF expression is also significantly decreased, likely due to 5-HT receptor dysregulation [61, 62]. As expected, Myelophil treatment significantly attenuated alterations in BDNF-TrkB signaling molecules, including BDNF itself and the expressions of CREB and c-Fos proteins (Fig. 5A to C). In an experiment with adult mice, depletion of TrkB specifically in 5-HTergic neurons led to excessive 5-HT production [63]. Serum BDNF levels in ME/CFS patients were shown to be reduced by about 60% compared to healthy controls [64].

Based on our results, Myelophil demonstrates clinical relevance for anti-central fatigue effects. In this study, we selected Myelophil doses of 50 and 100 mg/kg based on clinical usage (typically 2–4 g for adults) [28]. However, only the 100 mg/kg dose showed statistically significant effects. The 50 mg/kg dose did not reach statistical significance, similar to ascorbic acid (a positive control agent), which is widely used for fatigue associated with muscle dysfunction [65, 66]. While these findings provide the first experimental evidence that Myelophil can improve central 5-HTergic fatigue, several limitations remain. We currently do not know which specific components of Myelophil are primarily responsible for its effects. Further studies incorporating detailed electrophysiological and microhistological analyses are necessary to directly confirm Myelophil’s effects on serotonin spillover-induced motor dysfunction. Additionally, a direct comparison with serotonin-inhibitory agents should be performed.

Taken together, these findings suggest that Myelophil alleviates central fatigue, particularly in cases involving hyper-serotonergic activity in the brain. Our data support experimental evidence that may help elucidate the pharmacological basis of Myelophil’s potential benefit in conditions associated with central fatigue, such as CFS.

## Electronic supplementary material

Below is the link to the electronic supplementary material.

Supplementary Material 1

Supplementary Material 2

Supplementary Material 3

## Data Availability

The datasets utilized and/or analyzed in the present study are available from the corresponding author upon reasonably requesting them.
