# Supplementary figures and images for: Anti-central fatigue effects of myelophil in 5-HTergic hyperactivity mice model
Source: BMC Complement Med Ther. 2025 Apr 23;25:153. doi: 10.1186/s12906-025-04882-2 (PMC12020330; doi:10.1186/s12906-025-04882-2)

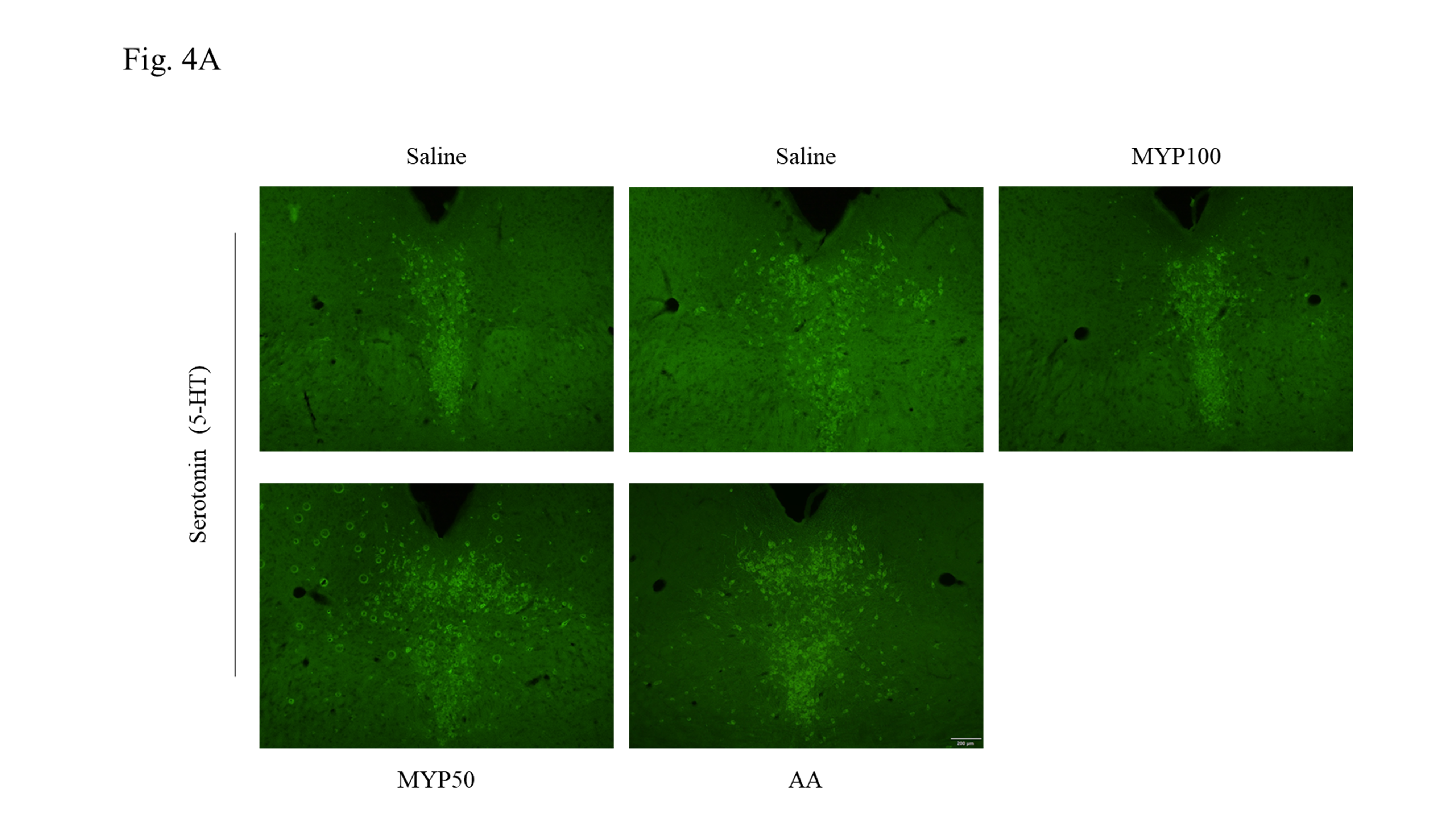

Supplement: Supplementary file 1 — Supplementary Material 1 [file 12906_2025_4882_MOESM1_ESM.tif]

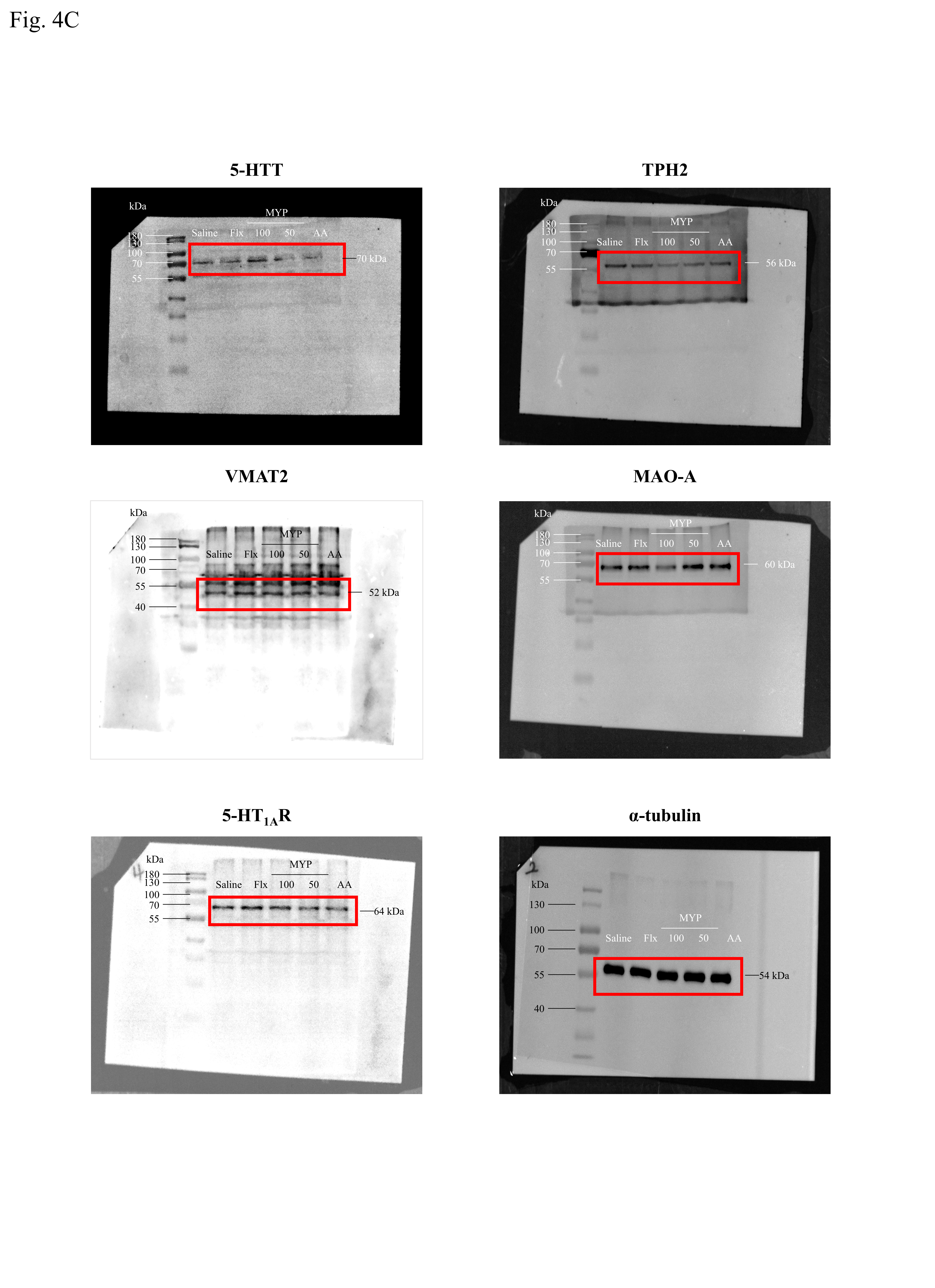

Supplement: Supplementary file 2 — Supplementary Material 2 [file 12906_2025_4882_MOESM2_ESM.tif]

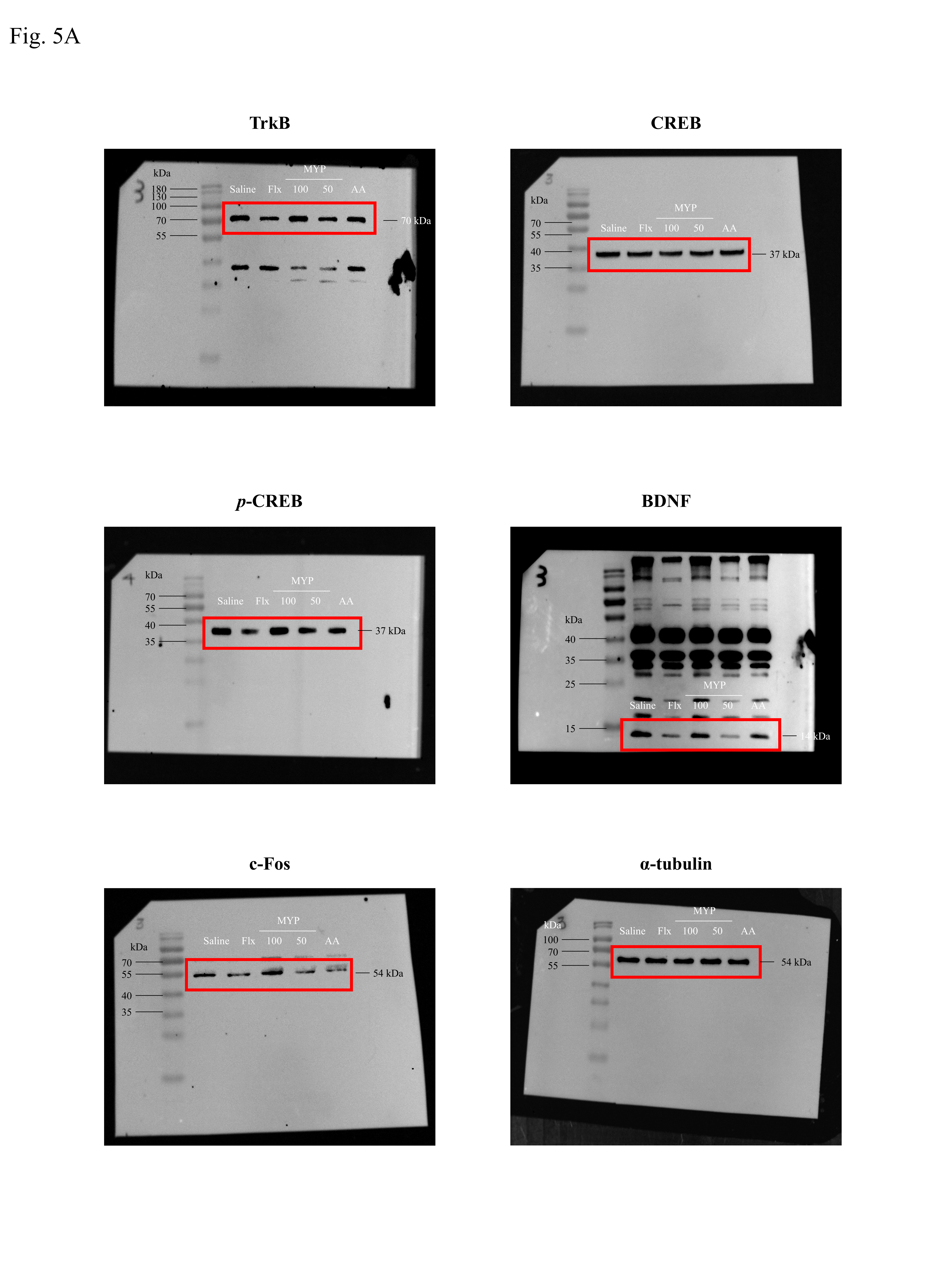

Supplement: Supplementary file 3 — Supplementary Material 3 [file 12906_2025_4882_MOESM3_ESM.tif]
